# Contextualization of Diabetes: A Review of Reviews from Organisation for Economic Co-operation and Development (OECD) Countries

**DOI:** 10.1007/s11892-024-01574-y

**Published:** 2025-01-24

**Authors:** Sieara Plebon-Huff, Hubi Haji-Mohamed, Helene Gardiner, Samantha Ghanem, Jessica Koh, Allana G. LeBlanc

**Affiliations:** Centre for Surveillance and Applied Research, Health Promotion and Chronic Disease Prevention Branch, Public Health Agency of Canada, 785 Carling Ave, Ottawa, Ontario K1A 0K9 Canada

**Keywords:** Systematic review, Contextual factors, Diabetes, Surveillance

## Abstract

**Purpose of Review:**

The prevalence of diabetes is rising around the world and represents an important public health concern. Unlike individual-level risk and protective factors related to the etiology of diabetes, contextual risk factors have been much less studied. Identification of contextual factors related to the risk of type 1 and type 2 diabetes in Organisation for Economic Co-operation and Development (OECD) countries may help health professionals, researchers, and policymakers to improve surveillance, develop policies and programs, and allocate funding.

**Recent Findings:**

Among 4,470 potential articles, 48 were included in this review. All reviews were published in English between 2005 and 2023 and were conducted in over 20 different countries. This review identified ten upstream contextual risk factors related to type 1 and type 2 diabetes risk, including income, employment, education, immigration, race/ethnicity, geography, rural/urban status, built environment, environmental pollution, and food security/environment.

**Summary:**

The ten upstream contextual risk factors identified this review may be integrated into diabetes research, surveillance and prevention activities to help promote better outcomes for people at risk or living with diabetes in OECD countries. Additional research is needed to better quantify the measures of associations between emerging key contextual factors and diabetes outcomes.

## Introduction

Prevalence of diabetes has risen around the world and represents an important public health concern. While there is no cure, advances in pharmacotherapy and behavioural interventions have improved management such that people are living longer healthier lives with diabetes. Globally, approximately 10.5% of adults were living with diabetes in 2021, and this is expected to rise to 12.2% by 2045 [1]. The Organisation for Economic Co-operation and Development (OECD) is an international organisation collaborating with its 38 member countries who share similar economic structures and health system frameworks to establish international standards on a variety of key global issues, including improving public health. On average, across OECD countries, 6.9% of adults were living with diabetes in 2021, with age-standardized prevalence rates exceeding higher than 10% in some countries [2].

Approximately 90% of all diabetes diagnoses are type 2 diabetes (T2D), 9% are type 1 diabetes (T1D), and 1% are other types of diabetes [3]. T2D results from a wide range of modifiable and non-modifiable individual, social, and ecological factors. It can often be prevented or delayed and commonly affects adults [4, 5]. Conversely, T1D is an autoimmune disease, which cannot be prevented. Other types of diabetes include gestational diabetes, and types secondary to gene mutations, medications, or other medical conditions [5].

The risk and management of diabetes are affected by a combination of individual characteristics and behaviours, and upstream contextual factors stemming from the social, economic, and physical environments [6]. Individual-level risk factors have been extensively studied and often concentrate on downstream aspects of health care, while upstream contextual factors are not well understood. Examples of upstream contextual factors include built environment, socioeconomic status, and culture [7]. A recent study examining geographic areas with high diabetes related mortality described these clusters as being less economically favourable, with high rates of unemployment and poor access to social services [8]. Moreover, Cunningham et al. [9] reported that 42% of the variation in diabetes incidence across the U.S. could be explained by socioeconomic and health inequalities such as higher unemployment rates, higher poverty rates, and longer commute times. A better understanding of upstream contextual factors related to diabetes risk is important to inform effective surveillance systems, public health policies, and programs to help reduce the risk of diabetes and improve the lives of those with the condition [10]. Thus, the purpose of this systematic review of reviews was to summarize the impact of upstream contextual factors related to the risk of T1D and T2D among OECD countries and to enhance diabetes surveillance activities through the application of health equity principles. We focused our review on OECD countries as they share a common goal of improving population health through a large range of determinants of health including upstream contextual factors [2], leading to a potentially greater amount of research on the topic.

## Methods

### Design

Registration of this systematic review was performed through the International Prospective Register of Systematic Review (PROSPERO registration number: CRD42020148159) in adherence to the Population, Exposure, Comparisons, Outcomes, and Study design framework (PECO) [11] (see Table 1), and the Preferred Reporting Items for Systematic Reviews and Meta-Analyses (PRISMA) guidelines [12].

**Table 1:** PECO criteria

Population	Review must include no more than 30% of publications from non-OECD countries. No exclusions with respect to age, gender, or ethnicity.
Exposure	Any contextual variables associated with diabetes (physical, sociocultural, political and economic environments).
Comparator	None required
Outcomes	Measures of association:
	• Risk ratio (RR): The risk of diabetes in the exposed group compared to the unexposed group.
	• Odds ratio (OR): The odds of diabetes in the exposed group compared to the unexposed group.
	Descriptive Measures:
	• Prevalence of diabetes (total proportion of cases in a population)
	• Incidence of diabetes (number of new cases, as a proportion or rate, in a given time period)

### Search Strategy

The search strategy was developed in partnership with the Health Library of Health Canada [13]. Embase and Medline were systematically searched using key words related to diabetes risk (e.g., diabetes incidence, diabetes prevalence) in OECD countries. Any type of review (e.g., systematic, meta-analysis, narrative) that reported on rates of diabetes as they relate to contextual factors were eligible for inclusion. The term ‘contextual factor’ was defined as physical, societal, and environmental factors that are associated with health outcomes. Reviews presenting data exclusively on individual factors (e.g., genetic, individual wealth) related to diabetes risk, diabetes treatment or diagnosis practices, reviews from non-OECD countries, unpublished reviews, conference abstracts, and reviews published in languages other than English or French were excluded. To ensure factors were relevant to current policy considerations, only reviews published since 1999 were eligible for inclusion. Within a given review, there were no date restrictions, and no exclusions with respect to characteristics of study participants, besides the fact that over 70% needed to be from OECD countries. Only reviews presenting data exclusively on T1D and/or T2D were included. All records were imported to RefWorks software where duplicates were removed. The search was completed on June 12, 2023.

### Study Selection

Four reviewers (AL, SPH, HH, HG) screened titles and abstracts using Covidence software (Veritas Health Innovation Ltd, Australia) and Microsoft Excel 2010. Reviews identified as potentially relevant by at least one reviewer were retrieved for full-text evaluation. Discrepancies were resolved by consensus.

### Data Extraction

Data were extracted in Microsoft Excel 2010 by three reviewers (SPH, HH, HG), using a standardized and pilot-tested data collection tool. Information extracted included study author(s), publication date, study design, study country, number of primary articles included in review, study population, diabetes type(s), and contextual factor(s). Reviewers were not blinded to study details.

### Data Synthesis

A narrative synthesis of each review was conducted. Where possible, study statistics were reported as prevalence (%), summary statistic (i.e. RR, OR), including 95% confidence intervals (CI). Each study could include multiple contextual factors.

### Quality Assessment

Systematic reviews were scored with the AMSTAR 2 tool [14] as high quality (no, or one non-critical weakness), moderate quality (more than one non-critical weakness), low quality (one critical weakness, or several non-critical weaknesses), or critically low quality (more than one critical flaw with or without non-critical weaknesses). Narrative reviews or papers without clear methods selection were evaluated with the SANRA tool [15] as high quality (score ≥ 10), moderate quality (7 ≤ score < 10), low quality (4 ≤ score < 9) or critically low quality (score < 4).

## Results

### Review Selection

**Fig. 1:**
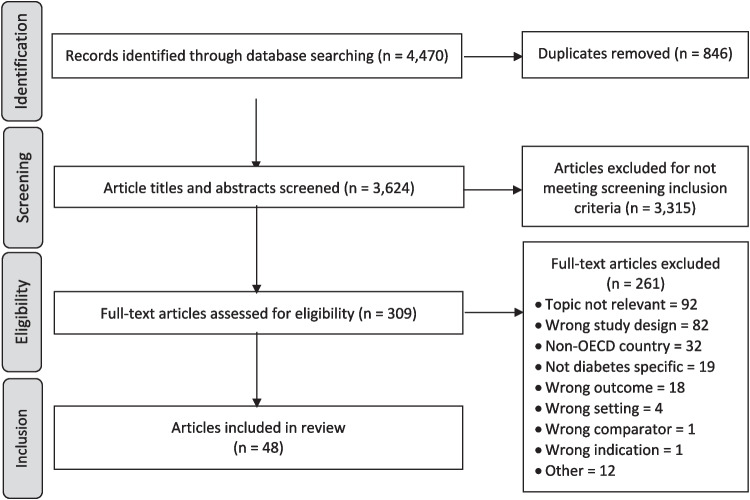
PRISMA flow diagram of articles included in review

After de-duplication, the search yielded 3,624 articles. A total of 309 were retained for full-text review and 48 were included in this overview (Fig. 1). Characteristics of included reviews are available in Table 2.

**Table 2:** Overview of included reviews

Source	Type of review (# primary studies included in the review)	Diabetes type	Study population(s)	Contextual factor(s)	Quality assessment (AMSTAR 2 or SANRA rating)
Adeghate et al. (2006) [37]	Narrative review	T1D, T2D	No specific focus	Race/ethnicity, Rural vs. urban status environmental pollution, food security/environment	Low
Adhikari et al. (2012) [28]	Systematic review (*n* = 15)	T1D, T2D	Canadian immigrants	Immigration, race/ethnicity	Critically low
Agardh et al. (2011) [16]	Systematic review and meta-analysis (*n* = 23)	T2D	No specific focus	Income, employment, education	Low
Aguayo-Mazzucato et al. (2019) [21]	Narrative review	T2D	Latino and Hispanic people	Income, education, immigration	High
Agyemang et al. (2009) [40]	Narrative review	T2D	People of Sub-Saharan African descent living in Europe	Race/ethnicity	Moderate
Agyemang et al. (2021) [52]	Narrative review	T2D	Migrants living in: Denmark, England and Wales, France, the Netherlands, Scotland, and Spain.	Race/ethnicity, built environment, food security/environment	Moderate
Bhupathiraju et al. (2016) [41]	Narrative review	T2D	US populations	Race/ethnicity	Low
Borchers et al. (2010) [17]	Narrative review	T1D	Children to adults under 40 years of age	Income, race/ethnicity, rural vs. urban status	Low
Castro et al. (2009) [19]	Narrative review	T2D	US Latino and other racial/ethnic populations	Income, immigration, built environment, food security/environment	High
Catherine et al. (2021) [24]	Narrative review	T1D, T2D	Children aged 0–19 from UK, US	Income	Moderate
Cheran et al. (2023) [44]	Systematic review (*n* = 13)	T2D	Indigenous populations in Canada	Race/ethnicity	Low
Dabelea et al. (2021) [39]	Review	T1D, T2D	Diabetes among 0–19 year olds from the US	Race/ethnicity	Moderate
Danaei et al. (2011) [46]	Meta-analysis (*n* = 128)	T1D, T2D	Adults 25+	Income, geography	Critically low
Den Braver et al. (2018) [51]	Systematic review (*n* = 109)	T2D	No specific focus	Rural vs. urban status, food security/environment	Moderate
Di Ciaula et al. (2021) [57]	Systematic review(*n* = 19)	T1D	Population aged 0–15 years in Europe	Environmental pollution	Critically low
Durazo et al. (2016) [45]	Narrative review	T2D	Latino population in the US	Race/ethnicity, built environment, food security/environment	High
Fayfman et al. (2017) [33]	Narrative review	T2D	Racial and ethnic minorities in the US	Immigration, race/ethnicity, food security/environment	Moderate
Ferrie et al. (2016) [25]	Meta-analysis (*n* = 19)	T2D	Employed individuals living in Australia, Denmark, Finland, Sweden, UK and US	Employment	Critically low
Goff et al. (2019) [32]	Narrative review	T2D	UK migrants	Immigration, race/ethnicity, urban vs. rural status	High
Gomber et al. (2022) [49]	Systematic review (*n* = 237)	T1D	Worldwide	Geography	Moderate
Gucciardi et al. (2014) [59]	Systematic review (*n* = 39)	T2D	North America	Food security/environment	Critically low
Ismail et al. (2022) [35]	Systematic review (*n* = 10)	T2D	UK migrants	Race/Ethnicity	Critically low
Kuo et al. (2013) [54]	Systematic review (*n* = 29)	T2D	No specific focus	Environmental pollution	Critically low
Meo et al. (2022) [53]	Systematic review and meta-analysis (*n* = 16)	T2D	China, Canada, US, Australia, UK, Hong Kong, Korea, Belgium, Bangladesh	Built environment	Critically low
Maier et al. (2013) [20]	Meta-analysis (pooled analysis) (*n* = 5)	T2D	German national population survey data	Income, education	Critically low
Martinez-Cardoso et al. (2020) [23]	Narrative review	Not specified	Immigrants living in US	Income, employment, education, immigration, built environment	Moderate
Meeks et al. (2016) [38]	Systematic review and meta-analysis (*n* = 20)	T2D	Ethnic minority populations in Europe	Race/ethnicity	Critically low
Minges et al. (2011) [43]	Systematic review (*n* = 24)	T1D, T2D	Indigenous populations in Australia	Race/ethnicity, rural vs. urban status	Critically low
Montesi et al. (2016) [31]	Narrative review	T2D	Migrants and ethnic minorities	Immigration, rural vs. urban status	Moderate
Neuenschwander et al. (2020) [60]	Systematic review and meta-analysis (*n* = 23)	T2D	US, Europe, Asia, Australia,	Food security/environment	Low
Oldroyd et al. (2005) [26]	Narrative review	T1D, T2D	Ethnic minority groups	Employment, race/ethnicity	Low
Park et al. (2014) [63]	Systematic review (*n* = 21)	T2D	No specific focus	Environmental pollution	Critically low
Pearce et al. (2021) [64]	Meta-analysis (*n* = 27)	T2D	USA, France, UK, Netherlands, Sweden Switzerland, Italy, Spain, Australia, Korea, Finland, Germany, Iran, China, Puerto Rico	Food security/environment	Critically Low
Perez-Escamilla et al. (2007) [34]	Systematic review (*n* = 27)	T2D	Latinos in the US	Immigration	Critically low
Rathmann et al. (2013) [18]	Narrative review	T2D	German adults	Income, environmental pollution	Moderate
Ricci-Cabello et al. (2010) [61]	Systematic review (*n* = 25)	T1D, T2D	OECD countries with universal healthcare	Income	Low
Sharp et al. (2009) [55]	Narrative review	T2D	Aboriginal peoples in Canada	Environmental pollution	Low
Testa et al. (2016) [30]	Narrative review	T2D	European migrants	Immigration, food security/environment	Low
Thibault et al. (2016) [27]	Systematic review (*n* = 77)	T2D	Canadian population	Income, education, rural vs. urban status, environmental pollution	Critically low
Vanderniet et al. (2022) [48]	Narrative review	T1D	Worldwide	Geography	Moderate
Wandell et al. (2010) [29]	Systematic review (*n* = 17)	T2D	Denmark, Finland, Iceland, Norway, Sweden	Immigration	Critically low
Wang et al. (2020) [56]	Meta-analysis (*n* = 8)	Not specified	Denmark, Switzerland, South Korea, Canada, Sweden, European Union	Environmental pollution	Low
Wedekind et al. (2021)	Narrative review	T2D	Indigenous populations in the US	Race/ethnicity	High
Weisman et al. (2018) [22]	Narrative review	T2D	Canada	Income, education, race/ethnicity, rural vs. urban status, food security/environment	High
Wu et al. (2022) [ 47]	Systematic review (*n* = 25)	T2D	Youth	Geography	Moderate
Yang et al. (2020) [58]	Meta-analysis (*n* = 16)	T2D	USA, Canada, Italy, Switzerland, Denmark, Germany, Hong Kong	Environmental pollution	Critically low
Yang et al. (2021) [62]	Narrative review	T2D	South Korean youth	Race/ethnicity, environmental pollution	Moderate
Zabetian et al. (2014) [50]	Systematic review, meta-analysis (*n* = 109)	T2D	Rural regions of high and low/middle income countries	Income, rural vs. urban status	Critically low

### Quality Assessment

Systematic reviews and meta-analyses (*n* = 26) were evaluated with the AMSTAR 2 tool. Almost all (*n* = 18) were assessed as critically low quality, five as low quality and three as moderate quality. Narrative reviews or reviews with no clear methods (*n* = 22) were evaluated using the SANRA tool. Five ranked as high quality, ten as moderate and seven as low quality. Quality assessment of included reviews are available in Table 2.

### Summary of Findings

Key contextual factors identified in the literature included income, employment, education, immigration, race/ethnicity, geography, rural/urban status, built environment, environmental pollution, as well as food security/environment.

A summary of findings by contextual factor of interest is included in Table 3.

**Table 3:** Summary of findings from included reviews

Contextual factor	# of reviews (# of systematic reviews or meta-analysis)	Summary of findings
Income	*n* = 13(6)	Inverse association between income and incidence and prevalence rates of diabetes, such that diabetes disproportionately affects those in lower income groups [16, 17, 18, 19, 21, 22, 61, 23, 20, 27, 24, 50, 46].
Employment	*n* = 4(2)	Incidence of diabetes was higher among those who reported high job insecurity and among low occupation groups [16,23,25,26].
Education	*n* = 6(3)	Significant inverse relationship between education and prevalence of diabetes, although this may be at least partially mediated by income [16,21,22,23,20,27].
Immigration	*n* = 10(3)	Recent immigration, and increased level of acculturation are associated with increased prevalence of diabetes. This may be mediated by income of native country [19,21,23,28,34,29,31,32,33,30].
Race and ethnicity	*n* = 17(4)	Incidence and prevalence of both T1D, and T2D are higher among some race and ethnic groups [17,22,26,32,33,43,38,37,39,40,41,45,52,28,62,42,^44^].
Geography	*n* = 4(2)	Most adults living with T2D were from China, India, USA, Russia, Indonesia, Japan, and Mexico [46]. Highest rates of T2D in youth were in the Western Pacific Region and in the World Bank upper-middle-income countries [47]. T1D incidence is highest in countries furthest from the Ecuador [48,49]
Rural and urban status	*n* = 9(4)	For T1D, decreased population density is associated with increased rate of disease, although this may be dependent of geographical location. For T2D, rates of diabetes are higher in more rural areas and in high density urban areas among at risk ethnic populations, although this may be mediated by socioeconomic status, genetics, or both [17,50,31,43,37,27,51,22,32].
Built environment	*n* = 5(1)	Evidence suggesting that an environment that promotes walkability, access to greenspaces, and safe and connected streets is associated with lower levels of obesity and higher levels of physical activity, which in turn may impact rates of diabetes [19,23,45,52,53].
Environmental pollution	*n* = 10(6)	Evidence suggesting that some environmental pollutants (air, food, and noise) are related to increased incidence and prevalence of diabetes, but this evidence is not definitive [18,37,27,54,63,57,55,56,58].
Food security and environment	*n* = 11(4)	Inverse relationship between diet quality and prevalence rates of diabetes. Food insecurity also plays and important role in the risk of diabetes [22,30,33,37,45,51,64,60,59,19,52].

### Socioeconomic Status

Socioeconomic status, measured via income, employment, or education, was consistently associated with increased risk of T2D. Agardh et al. [16] reported an overall increased risk of T2D in low socioeconomic groups whether measured by income (40%), education level (41%), or occupation (31%). Although there is considerable overlap between income, employment, and education, results are presented separately as much as possible.

#### Income (*n* = 13)

Borchers et al. [17] reported that rates of T1D vary considerably but incidence is lowest in areas of highest material deprivation. High population density and household crowding provided a better predictive model than global measures of socioeconomic deprivation. Incidence of T1D correlates most strongly with indicators of national prosperity such as gross domestic product and low infant mortality, although these results may be at least partially influenced by differences between urban and rural areas which may only serve as proxies for environmental and lifestyle factors.

Results from reviews focused on T2D suggest an increased risk of diabetes in the lower income groups. At the regional level, Rathmann and colleagues [18] reported that the unemployment rate and the regional economic climate of the governing body was associated with T2D prevalence. Using an Index of Multiple Deprivation, the DIAB-CORE consortium confirmed that diabetes is more prevalent in economically weaker regions and increases with unemployment and poorly built environment [18]. Castro et al. [19] suggests that environmental factors such as impoverished neighborhoods are an important modifiable risk factor for T2D. Maier et al. [20] found that the risk of T2D was greater in lowest income groups compared to highest (OR 2.08, 95% CI 1.63–2.65), and in most deprived groups, compared to the least deprived (OR 2.43, 95% CI 1.47–4.04). In sub-group analysis by income level of high-income countries, low education level and low-income occupation level were associated with a 45% and a 31% increased risk of T2D, respectively [16]. Poverty or low socioeconomic status was found to increase the risk of incident T2D and related complications [21]. Data from a Canadian study illustrated a 14% higher incidence of T2D in Ontario in the lowest income quintile relative to the highest (8.26 per 1,000 vs. 7.25 per 1,000), with consistent findings across all ages and sexes [22]. Some populations are overrepresented in lower paying job sectors (e.g., immigrants, ethnic minorities) and are at greater risk of deprivation, food insecurity, diabetes incidence, sub-optimal diabetes management, and poor glycemic control [23, 24].

#### Employment (*n* = 4)

Ferrie et al. [25] reported that high job insecurity at baseline was associated with an increased risk of T2D in the age- and sex-adjusted analysis compared with low job insecurity (OR 1.19, 95% CI 1.09–1.30). This also remained significant after adjusting for socioeconomic status, obesity, physical activity, smoking, and alcohol use (OR 1.12, 95% CI 1.01–1.24). Oldroyd et al. [26] reported higher age- and sex-standardized mortality rates for T1D and T2D in manual workers than in non-manual workers, independently of socioeconomic position. Based on income level of occupation, the lowest earning employment groups showed highest risk of T2D compared to the highest earning employment groups (RR 1.31, 95% CI 1.09–1.57) [16]. Martinez-Carsodo et al. [23] also reported that immigrants account for 30% of the uninsured population and tend to work in sectors without healthcare access. Thus, they are less likely to use healthcare, and are at increased risk of having undiagnosed (OR 3.74, 95% CI 2.29–6.08) or uncontrolled diabetes (OR 1.94, 95 CI 1.31–2.88), compared to non-immigrant populations.

#### Education (*n* = 6)

All included reviews reported a significant inverse relationship between education and risk of diabetes [16, 20–23, 27]. For example, among Hispanics living in the US, prevalence of diabetes was 15% for those with less than a high school diploma, compared to only 7% among those with a bachelor’s degree or higher [21]. Agardh et al. [26] showed that T2D risk was significantly higher in the lowest, compared to the highest education group (RR 1.41, 95% CI 1.28–1.51). Similarly, Maier et al. [20] reported an increased risk of diabetes for those with low compared to medium/high education levels (OR 1.99, 95% CI 1.71–2.32).

### Immigration, Race and Ethnicity

In many cases, it was difficult to disentangle discussion about immigration, race and ethnicity due to the inherent complexity of these terms and confounding variables. Often, all three concepts were used to calculate acculturation index, which are delineated in the next sections. These concepts were defined by the authors of each review to reflect the unique racial and ethnic composition of their study population, shaped by the region’s historical context, current sociopolitical landscape, and geographical characteristics.

#### Immigration (*n* = 10)

In most reviews, immigration was defined as time since arrival, country of origin, parents’ country of origin, or ethnic background. Overall, it appeared that time since arrival was correlated with prevalence of diabetes. Adhikari et al. [28] reported a higher prevalence of diabetes among immigrants living in Canada for 15 years or more (male OR 1.52; female OR 1.40) compared to residents of 5 to 9 years. In Denmark, Finland, Iceland, and Norway, prevalence of T2D was highest among immigrants of non-OECD countries, followed by immigrants of OECD countries; non-immigrants had the lowest rates of diabetes [29]. Results were similar from other reviews with higher reports of diabetes prevalence and incidence rates among non-European migrants compared to European born people [30–32]. Length of residence in the US was positively correlated with prevalence of T2D, suggesting that immigrants’ health worsens with time spent in the US [23, 33].

Acculturation, or the level to which immigrants adopt attitudes, values, customs, beliefs, and behaviours of a new culture, may also help to explain disease risk [34]. Acculturation is often calculated as an index based on proxy indicators such as birthplace, language use, and number of years spent in a new country. Low levels of acculturation were associated with better health, including lower rates of T2D among Latinos in the US [34]. Based on results from the National Health and Nutrition Examination Study in the US, Aguayo-Mazzucato et al. [21] reported increased risk of T2D corresponding to the level of acculturation with scores ranging from 0 (lowest) to 3 (highest). After adjusting for socio-demographic factors, the study found an increased risk of T2D for acculturation scores of 1 (OR 1.71, 95% CI 1.31–2.23), of 2 (OR 1.63, 95% CI 1.11–2.39) and of 3 (OR 2.05, 95% CI 1.27–3.29) compared to those with a score of 0. Also, Castro et al. [19] reported that T2D prevalence was significantly associated with acculturation in non-Mexican Latinos, but not in Mexican-origin Latinos nor in people of Chinese origin. Finally, English proficiency among Indian and Bangladeshi UK migrants was associated with a lower prevalence of diagnosed and undiagnosed diabetes [35].

#### Race and Ethnicity (*n* = 17)

Although sometimes used interchangeably, race categorizes people solely based on their physical characteristics, while ethnicity is a multi-dimensional concept that categorizes people based on their culture, religion, language, nationality, geographic origin, etc [36]. Highest rates of T1D were found in Nordic countries and lowest rates were found in Asian, Caribbean, and southern European countries [37]. Outside of Nordic countries, prevalence of T1D seems to be highest among non-Hispanic whites compared to other ethnic groups. This may be due to European ancestry of this group in the US, Canada, Australia, and New Zealand [17].

On the other hand, T2D prevalence was two to three times higher among minority ethnic groups in the UK, compared to the white British population [32]. Moreover, several reviews have shown rates of T2D to be lowest in non-Hispanic whites and highest among South Asians [22, 26, 28, 33, 38–41]. For example, in a pooled analysis, Meeks et al. [38] reported that compared to people of European descent, South Asians had the highest risk of T2D (OR 3.7, 95% CI 2.7–5.1), followed by people of Middle Eastern and North African, Sub-Saharan African, Western Pacific, and South and Central American descent. According to Agyemang et al. [40], migrants living in Europe have an increased risk of T2D compared to the general population: South Asia (OR 3.7, CI 95% 2.7–5.1); Middle East (OR 2.7, CI 95%1.8–3.9); North Africa, Sub-Saharan Africa (OR 2.6, CI 95% 2.0–3.5) and South and Central America (OR 1.3, CI 95% 1.1–1.6). The prevalence of type 2 diabetes was also higher among Indigenous people in Canada [22, 28], in the United-States [42] and in Australia [43] compared to other ethnic groups. Multiple and complex contextual factors have been contributing to the increased risk of diabetes in the indigenous population in Canada, including the impact of centuries of colonization and marginalization, and other environmental, sociocultural, socioeconomic, and biological/genetic factors [44]. Durazo et al. [45] examined the neighbourhood concentration of race and ethnic minority groups and although results were mixed, some studies indicated that residents of ethnic enclaves exhibit better health than those living outside.

### Environment

#### Geography (*n* = 4)

The burden of T2D was found to increase generally in countries with a growth and ageing population. Most adults living with T2D were from China and India (40%), USA and Russia (10%), and Indonesia, Japan, and Mexico (12%) [46]. Most youth living with T2D were from the Western Pacific Region (30%) and from the World Bank upper-middle-income countries (40%). Countries with highest incidence of T2D in youth were China, India, and United States [47]. T1D rates were found to be highest in countries furthest from the equator such as Northern Europe Australia, New Zealand, Northern America and Northern Africa [48, 49].

#### Rural and Urban Status (*n* = 9)

Borchers et al. [17] found that T1D prevalence was highest in areas with lower population density, such as rural areas. For T2D, evidence shows that rural areas tend to have higher rates compared to urban areas, although this may be related partially to socioeconomic status and the presence of risk factors for diabetes, like obesity [31, 37, 50]. A few authors [22, 31] suggest that economic development and rapid urbanization in low to middle-income countries have contributed to increasing rates of diabetes. Within Canada, they reported geographic variation, with the lowest rates in Western Canada (British Columbia, 5.5%) and the highest rates in Newfoundland (9.0%). Minges et al. [43] reported, based on all three studies they included, diabetes prevalence in indigenous Australian populations living in remote area was approximately twice that in the urban populations. Finally, after stratifying by country-level income, a meta-analysis of 19 studies [51] showed that the risk of T2D in urban versus rural areas was not significantly different (OR 1.15, 95% CI 0.70–1.89), however, highly walkable neighbourhoods were associated with a decreased in risk (OR 0.79, 95% CI 0.71–0.87).

#### Built Environment (*n* = 5)

Limited evidence from four narrative reviews suggested that an environment promoting walkability, access to greenspaces and safe and connected streets is associated with lower levels of obesity and higher levels of physical activity, which in turn impact rates of T2D [19, 23, 45, 52]. Neighbourhood characteristics like density of healthy food outlets or fast-food outlets, crime, perceived safety, walkability and access to parks/green space was found to impact insulin resistance and diabetes through their influence on physical activity and diet [45]. A meta-analysis of 13 studies concluded that green residential environment was associated with a decrease in T2D prevalence (OR 0.88, 95% CI 0.86–0.89) and mortality (HR 0.92, 95% CI 0.90–0.93) [53].

#### Environmental Pollution (*n* = 10)

Limited evidence shows that environmental pollutants in food sources may be associated with an increased risk of diabetes. High intake of nitrites and n-nitroso compounds were associated with T1D in children [37]. Kuo et al. [54] also noted a causal association between T2D and the presence of arsenic, mercury, cadmium, persistent organic pollutants (POP), phthalates, and bisphenol A (BPA) in foods. Consuming contaminated water and foods can result in the bioaccumulation of environmental toxins in the food chain like arsenic, mercury, diocin-like compounds, phalates, and BPA [55]. These contaminants can interfere with pancreas function and disrupt the endocrine system leading to obesity, a known risk factor for diabetes. Indigenous people in Canada experience 3–5 times higher rates of diabetes compared to the general population; this disparity may be partially explained by the bioaccumulation of toxins in traditional diet (wild game and fish) [55].

In general, there were limited details on measurement of environmental pollutants, but many studies included satellite images of pollution level in combination with health administrative data sources. Air-borne pollutants result from many [56] mitigatable contextual sources, including but not limited to forest fires, road traffic, manufacturing industries, and in home settings. For example, Thibault et al.’s [27] systematic review focusing on Canadian population found that nitrogen dioxide (NO_2_), particulate matter (PM), POP, and non-persistent pesticides could partially explain recent increase in T2D prevalence. Also, prolonged exposure to NO_2_, PM2.5, and PM10 has been associated with increased risk of T2D [27, 57, 58]. Stratified analysis indicated that PM2.5 exposure was associated with an increase in T2D incidence in American countries, but not in European countries [58]. Di Ciaula and colleagues [57] found that T1D incidence among youth aged 0–15 years was greater in European countries with greater emissions of PM10, NO, and volatile organic compounds (VOCs).Wang et al. [56] found that noise pollution may also increase the odds of developing diabetes (OR 1.08; 95% CI 1.03–1.12).

#### Food Security/Environment (*n* = 11)

Upstream contextual factors contributing to poor diet includes food insecurity, and poor food environment characterised by limited access to healthy foods or access to an abundance of unhealthy foods. In general, an inverse relationship between diet quality and rates of T2D was observed. Poor diet was generally defined as energy dense and nutrient poor.

After controlling for income, employment status, and some physical/lifestyle factors, Gucciardi et al. [59] found that the prevalence of T2D was 2–3 times higher among adults who experience food insecurity in North America compared to those that are food secure. The prevalence of food insecurity was also higher among those with diabetes and the risk of food insecurity increased with length of diabetes diagnosis. More specifically, four reviews suggested that income plays an important role such that those experiencing food insecurity or living in food deserts are at increased risk of T2D [19, 33, 37, 60]. On the contrary, den Braver et al. [51], concluded that there is no statistically significant relationship between availability of supermarkets/grocery stores, or availability of fast-food outlets/convenience stores, and risk of T2D.

Adeghate et al. [37] reported that diets higher in fat, and lower in fibre are associated with obesity and T2D. Weisman et al. [22] indicated that consumption of high glycemic index foods and sugar-sweetened beverages are associated with increased risk of T2D, and that high fiber foods has a protective effect.

## Discussion

This overview aimed to identify physical, socio-cultural, environmental, political, and/or economic contextual factors related to the incidence or prevalence of T1D and T2D. This review identified 48 reviews, including 26 systematic reviews (with or without meta-analysis), and 22 narrative reviews.

Ten contextual factors related to diabetes were identified: income, employment, education, immigration, race/ethnicity, geography, built environment, environmental pollution, rural/urban status, and food security/environment.

Due to heterogeneous nature of reviews regarding population characteristics, study location, measures used, and outcomes reported, summary statistics could not be calculated. However, our results suggest that race/ethnicity and income may be the most important factors related to diabetes prevalence and that many of the identified contextual factors interact with each other and may be confounded by overlapping variables. For example, rural or urban status may be an important factor related to diabetes prevalence, but this may also be because those living in rural regions may belong to lower socioeconomic groups, and may be exposed to poor built environment, therefore putting them at higher risk for obesity and diabetes. These findings should be interpreted with caution since some primary studies may have not been in the included reviews or were over-represented due to overlap across reviews. Additionally, reviews identified both individual-level and ecological studies. More evidence amongst individuals with diabetes would be beneficial to strengthen current measures of association related to diabetes and upstream contextual factors.

Our initial protocol only included the AMSTAR 2 quality assessment tool, specifically designed for systematic reviews. Since almost half of the reviews were narrative, we had to incorporate a second quality assessment tool (SANRA). Most systematic reviews were of low or critically low quality, while most narrative reviews were ranked as moderate to low quality. Given that the World Health Organization identified diabetes as “one of the major public health challenges in the 21st century” [65] and considering the OECD’s highlights the influence of demographic, economic, and social contexts on health status and outcomes [2], it is surprising that there is lack of high-quality evidence from OECD countries. This further emphasizes the need for more studies of good quality providing qualitative evidence on contextual factors related to diabetes risk.

Despite the low quality and lack of qualitative evidence in the current literature, this work provides insights into some broader factors that may influence rates of diabetes that warrant further investigation and that could be incorporated into future diabetes surveillance reporting among OECD countries. Several reports [66–68], have called upon improvements of public health surveillance through the monitoring of social determinants of health to support health promotion, intervention programs, and chronic disease prevention. Therefore, health surveillance systems should go beyond traditional disease indicators, and incorporate context-dependent metrics to apply health equity principles. This broadens the system’s capacity to monitor intervention and policy effectiveness, offering potential insights into observed trends or patterns [69].

## Conclusion

In addition to shaping the values and relationships attributed to health, socioeconomic, cultural, political, and physical contexts also contribute to the availability, distribution and quality of public infrastructure and resources. These in turn impact key social determinants of health such as housing, education, social services, and healthcare that contribute to the burden of diabetes and other chronic diseases in OECD countries [10].This review identified ten provisional upstream contextual variables that should be further explored, including income, employment, education, immigration, race/ethnicity, geography, built environment, environmental pollution, rural/urban status, and food security. Thus, qualitative evidence on these factors at the individual level, including comparators without diabetes, can inform urban planning for a healthier living environment. Future research should delve into non-traditional diabetes risk factors like built environment, environmental pollution, and food security/environment to enhance surveillance and inform public health policies.

## Key references

1. Agyemang et al. (2021) [52] (very important): This review examines the high burden of type 2 diabetes among migrant populations in Europe, identifying complex causal pathways involving social, environmental, and healthcare-related factors. It highlights differences in type 2 diabetes rates across racial and ethnic groups, the influence of the built environment, and the role of food security in diabetes risk. These findings suggest that investigating structural inequalities is key to improving diabetes outcomes among migrant populations, and supports our argument for focusing on upstream factors in diabetes prevention.
2. Dabelea et al. (2021) [39] (very important): This review, drawing on twenty years of surveillance data, concluded that significant disparities exist in pediatric type 1 and type 2 diabetes rates across racial and ethnic groups. It highlights the importance of identifying key social determinants of health and emphasizes the need to consider these factors when shaping health policies, particularly during the transition from pediatric to adult care.
3. Gomber et al. (2022) [49] (important): This scoping review examines the global variation in T1D incidence, particularly among children and adolescents, with an emphasis on the variability in incidence rates of T1D by geographic region, income group, and age. The paper supports the exploration of geographical factors influencing diabetes outcomes.

## Data Availability

No datasets were generated or analysed during the current study.
